# 

**Published:** 1870-06

**Authors:** 


### CONTENTS.

ORIGINAL COMMUNICATIONS.

Alveolar Abscess, with Caries or Necrosis—Their Phenomena, Treatment, &c , &c.......... 241
Diseases and Treatment of the Dental Pulp.......... 258
Anæsthesia .......... 264

PROCEEDINGS OF SOCIETIES.

Northern Ohio Dental Association.......... 267
The American Dental Association.......... 271

SELECTIONS.

Opium and Belladonna.......... 273
Dr. Storer on Dr. Simpson.......... 275
Ether vs. Chloroform .......... 277
Hypodermic Injections.......... 278
Shut Your Mouth.......... 280
Obituary.......... 282

EDITORIAL.

Unmotherly.......... 283

